# Invasive *Listeria monocytogenes* Gastroenteritis Leading to Stupor, Bacteremia, Fever, and Diarrhea: A Rare Life-Threatening Condition

**DOI:** 10.1177/2324709617707978

**Published:** 2017-05-09

**Authors:** Hassan Mehmood, Asghar Dil Jan Khan Marwat, Noman Ahmed Jang Khan

**Affiliations:** 1 Department of Internal Medicine, Conemaugh Memorial Medical Center, Johnstown, PA, USA

**Keywords:** *Listeria monocytogenes*, invasive listeriosis, nonbloody diarrhea, altered mental status, ampicillin

## Abstract

*Listeria monocytogenes* is a gram-positive, rod-shaped organism that can cause serious infections such as meningitis, invasive gastroenteritis, and endocarditis. Every year 1600 people in the United States are affected, with significant mortality of 260 people annually. *Listeria* gastroenteritis has the third highest mortality rate among all the food-borne infection. Invasive listeriosis most commonly affect pregnant women, those in extremes of ages, people with comorbid diseases, and people with weakened immune response. In this article, we present a rare case of invasive *Listeria* gastroenteritis in an 83-year-old female with multiple comorbid conditions and past medical history of type 2 diabetes mellitus and multiple risk factors who was brought to the hospital with altered mental status. She had history of fever, abdominal pain, and watery diarrhea up to 14 episodes in 24 hours for the last 7 days. Her stool culture grew *Listeria monocytogenes* sensitive to penicillin. She was started on penicillin for 2 weeks. She had subsequently complete resolution of fever, diarrhea, and abdominal pain. High index of suspicion is the key to ensure timely initiation of appropriate empirical treatment in the setting of invasive gastroenteritis, especially in people who have high risk factors for listeriosis. We recommend raising awareness in the health care profession about invasive listeriosis in the need of time. Intravenous ampicillin or penicillin G is the treatment of choice, with meropenem as an alternative.

## Introduction

*Listeria monocytogenes* is a gram-positive, rod-shaped organism that can grow in aerobic and anaerobic conditions. Invasive listeriosis is rare in the United States, with an annual incidence of 1600 cases, resulting in 260 deaths annually.^1^ *Listeria* has the third highest mortality rate among all food- borne infections.^1^ *Listeria* is present in soil, water, some animal sources, raw (unpasteurized) milk, ready-to-eat deli meats, hot dogs, and refrigerated smoked seafood.^2^ Unlike common food-borne diseases, the incubation period can be long. Median incubation period is 8 days (range = 1-67 days).^3^ Proper cooking and pasteurization can kill *Listeria*. Invasive *Listeria* most commonly affects pregnant women; those in extremes of ages, that is, new born and people above 65; and people with comorbid diseases such as diabetes mellitus, immunosuppression, HIV, and liver disease. Listerial gastroenteritis is self-limiting in healthy individuals without any serious complications.^4,5^

## Case History

An 83-year-old female with past medical history of type 2 diabetes mellitus, ischemic heart disease, hypertension, hyperlipidemia, and chronic back pain was brought to the hospital with altered mental status and nonbloody diarrhea. On physical examination, she was febrile with a temperature of 38.9°C, blood pressure of 95/60 mm Hg, and pulse of 134 per minute. She was stuporous, lethargic, and unable to follow any commands with withdrawal to painful stimuli only. She had up to 14 episodes of diarrhea in last 24 hours. Initial laboratory work up was remarkable for white blood cell count of 23 000/µL, sodium of 130 mmol/L, bicarbonate of 16 mEq/L, lactate of 4.2 mmol/L, creatinine of 2.8 mg/dL, and blood glucose of 394 mg/dL. Computed tomography scan of abdomen and pelvis without contrast was performed, which showed some nonspecific findings of enteritis. Given the very high suspicion for *Clostridium difficile*, she was empirically started on intravenous metronidazole and oral vancomycin and stools were sent for *Clostridium difficile* toxin. Two days later her blood cultures grew *Listeria monocytogenes*, sensitive to penicillin, and stool came back negative for *Clostridium difficile* toxin. Intravenous metronidazole and oral vancomycin were stopped and subsequently penicillin was initiated. She responded well with complete resolution of diarrhea, abdominal pain, and fever.

## Discussion

*Listeria*, although an uncommon microorganism to affect the general population, is capable of causing a broad range of illness in neonates, pregnant women, elderly, and immunocompromised individuals. *Listeria monocytogenes* is a motile, non–spore-forming, gram-positive rod that has aerobic and facultatively anaerobic characteristics. Our patient’s advance age, history of diabetes mellitus, and other multiple comorbid conditions with suggestive clinical presentation were the clues for early suspicion of invasive *Listeria* gastroenteritis.^6^

The most common symptoms of invasive gastroenteritis due to *Listeria monocytogenes* are fever, diarrhea, arthromyalgia, and headache. In most cases, at least one gastrointestinal symptom such as nausea, vomiting, diarrhea, and abdominal pain is present. Diarrhea is typically nonbloody and watery stool with an average of 10 to 12 stools in 24 hours. Bloody diarrhea is rare and noted in only 3% of cases. Fatigue and sleepiness are present in 63% and 83% of cases, respectively.^7,8^ A rare complication of *Listeria* gastroenteritis is an invasive disease with positive blood culture, which can lead to severe sepsis and death.

Thirteen serotypes of *Listeria monocytogenes* are reported so far. Only serotypes 4b, 1/2a, and 1/2b are known to cause over 98% of the human listeriosis case reported.^9^ Serotype 4b is notorious for causing invasive listeriosis.^10^ Gastric acidity is protective against *Listeria* and people taking H2 antagonists have increased prevalence of asymptomatic *Listeria monocytogenes* in stool.^11^ Although *Listeria* does not produce any enterotoxin and the mechanism of pathogenesis is unknown, direct invasion of bacterial protein with E-cadherin on the host cell, leading to diarrhea, fever, and bacteremia, is proposed.^12,13^

The diagnosis of *Listeria* should be suspected based on high clinical suspicion. Stool culture is not recommended in systemic listeriosis; however, when investigating an outbreak of *Listeria*, special selective culture media should be used. The high titers of antibodies to listeriolysin O, the major virulence factor, can help in diagnosis, but blood culture is considered diagnostic for systemic listeriosis.^14^ In one study, antibodies to listeriolysin O was positive in 27 of 28 patients with invasive listeriosis, making it a useful study to discriminate between sporadic and invasive listeriosis. A new method of diagnosing amino-terminal residue of listeriolysin O may be more specific, but they are not commercially available yet.^15^

Treatment of invasive *Listeria* gastroenteritis is ampicillin 2 g intravenous every 4 to 6 hours for 2 weeks or penicillin G 4 million units intravenous every 4 hours, and the latter is considered the drug of choice.^16^ Patients allergic to penicillin should get skin test and desensitization if possible or use trimethoprim-sulfamethoxazole 3 to 5 mg/kg intravenous every 6 hours for 2 weeks. Noninvasive *Listeria* gastroenteritis is typically self-limiting, specifically in healthy young people with complete recovery in 2 to 3 days.

## Conclusion

Due to high mortality of invasive *Listeria* gastroenteritis, high degree of suspicion is the key to early diagnosis and initiation of proper treatment, especially in people in extreme of ages and multiple comorbid conditions wherein it presents with multiple episodes of diarrhea and altered mental status. We recommend raising awareness in the health care profession about invasive listeriosis in the need of time. Avoiding unpasteurized milk, thorough cooking of raw food from animal source, reheating hot dogs properly and deli meats, and hand hygiene can help in preventing transmission of infection from *Listeria monocytogenes*.
